# Association between lactic acidosis and multiple organ dysfunction syndrome after cardiopulmonary bypass

**DOI:** 10.7717/peerj.16769

**Published:** 2024-01-31

**Authors:** Dan Zheng, Guo-Liang Yu, Yi-Ping Zhou, Qiao-Min Zhang, Chun-Guo Wang, Sheng Zhang

**Affiliations:** 1 Department of Critical Care Medicine, Taizhou Hospital of Zhejiang Province, Wenzhou Medical University, Linhai, China; 2 Department of Cardiothoracic Surgery, Taizhou Hospital of Zhejiang Province, Wenzhou Medical University, Linhai, China

**Keywords:** Cardiopulmonary bypass, Acidosis, Lactic, Hyperlactatemia, Multiple organ dysfunction syndrome

## Abstract

**Background:**

The relationship between hyperlactatemia and prognosis after cardiopulmonary bypass (CPB) is controversial, and some studies ignore the presence of lactic acidosis in patients with severe hyperlactacemia. This study explored the association between lactic acidosis (LA) and the occurrence of multiple organ dysfunction syndrome (MODS) after cardiopulmonary bypass.

**Methods:**

This study was a *post hoc* analysis of patients who underwent cardiac surgery between February 2017 and August 2018 and participated in a prospective study at Taizhou Hospital. The data were collected at: ICU admission (H0), and 4, 8, 12, 24, and 48 h after admission. Blood lactate levels gradually increased after CPB, peaking at H8 and then gradually decreasing. The patients were grouped as LA, hyperlactatemia (HL), and normal control (NC) based on blood test results 8 h after ICU admission. Basic preoperative, perioperative, and postoperative conditions were compared between the three groups, as well as postoperative perfusion and oxygen metabolism indexes.

**Results:**

There were 22 (19%), 73 (64%), and 19 (17%) patients in the LA, HL, and NC groups, respectively. APACHE II (24h) and SOFA (24h) scores were the highest in the LA group (*P* < 0.05). ICU stay duration was the longest for the LA group (48.5 (42.5, 50) h), compared with the HL (27 (22, 48) h) and NC (27 (25, 46) h) groups (*P* = 0.012). The LA group had the highest incidence of MODS (36%), compared with the HL (14%) and NC (5%) groups (*P* = 0.015). In the LA group, the oxygen extraction ratio (O_2_ER) was lower (21.5 (17.05, 32.8)%) than in the HL (31.3 (24.8, 37.6)%) and the NC group (31.3 (29.0, 35.4) %) (*P* = 0.018). In the univariable analyses, patient age (OR = 1.054, 95% CI [1.003–1.109], *P* = 0.038), the LA group (*vs.* the NC group, (OR = 10.286, 95% CI [1.148–92.185], *P* = 0.037), and ΔPCO2 at H8 (OR = 1.197, 95% CI [1.022–1.401], *P* = 0.025) were risk factor of MODS after CPB.

**Conclusions:**

We speculated that there was correlation between lactic acidosis and MODS after CPB. In addition, LA should be monitored intensively after CPB.

## Introduction

Cardiac surgery comes with a significant risk of major adverse events, with an overall mortality rate of 2%–3% (Senst, Kumar & Diaz, 2022). Major complications include bleeding, stroke, kidney injury, mesenteric ischemia, atrial fibrillation, cardiogenic shock, and respiratory distress (Senst, Kumar & Diaz, 2022). Multiple organ dysfunction syndrome (MODS) occurs in about 5% of patients after cardiac surgery, with cardiopulmonary bypass (CPB) being the procedure with the highest risk (D’Agostino et al., 2018; Liu et al., 2021). The 28-day mortality rate of MODS is 23% (Stoppe et al., 2016), and MODS can persist for as long as 28 days in 15% of patients (Stoppe et al., 2016). MODS after cardiac surgery is associated with high morbidity and mortality. Predicting the risk of MODS after cardiac surgery could help screen patients requiring closer management.

Hyperlactatemia occurs in 10%–20% of patients following cardiac surgeries (Minton & Sidebotham, 2017), as it can result from hypoxic and nonhypoxic causes such as drug therapy, cardioplegia, hypothermia, and CPB. Severe hyperlactatemia can lead to lactic acidosis (LA; Garcia-Camacho et al., 2020; Greenwood et al., 2021; Teloh et al., 2017), which can then lead to vascular hyporeactivity, decreased peripheral vascular resistance, decreased myocardial contraction force, and reactivation of the heart and exogenous catecholamines, causing myocardial injury, severe arrhythmia, inflammation, and impaired immune cell function (Azad, Islam & Quasem, 2019; Kimmoun et al., 2015; Rudnick et al., 2020). The relationship between hyperlactatemia and prognosis after CPB remains controversial, however, and the limits of lactic acid values for hyperlactacemia are not uniform in published research (Joudi et al., 2014; Mak et al., 2016; Minton & Sidebotham, 2017). Normal lactate is generally defined as blood lactate <2.0 mmol/L (Foucher & Tubben, 2022), but because there is no standard definition for hyperlactatemia, it has been variably defined as blood lactate >3 or >4 mmol/L, or even >10 mmol/L (Bhardwaj et al., 2017; Kedziora et al., 2020; Renew et al., 2016). LA is a well-known marker of poor tissue perfusion and multiorgan dysfunction, and LA can predict mortality in patients with severe sepsis and septic shock and the incidence of MODS in hospitalized patients (Doshi et al., 2018; Gattinoni et al., 2019). LA and a longer time to lactic acid normalization are also associated with an increased risk of death (Bullock & Benham, 2022; Gillies et al., 2019; Radelfahr & Klopstock, 2019). However, there are few studies on the effect of LA on MODS after CPB. This study aimed to determine the association between LA and the occurrence of MODS after CPB. The results of this study could help improve the management of CPB patients.

## Materials & Methods

### Study design and participants

This study was a *post hoc* analysis of the clinical data from all patients who underwent cardiac surgery between February 2017 and August 2018 and participated in a prospective study (monitoring oxygen metabolism after cardiopulmonary bypass and 48-h organ dysfunction, ChiCTR-ROC-1701072; Zhang et al., 2021). This study was approved by the ethics committee of Taizhou Hospital (Zhejiang Province, China) and was carried out according to the tenets of the Helsinki Declaration of 1975 and good clinical practice. Written informed consent was obtained from all patients in the original study (Zhang et al., 2021), including consent to the use of study data for *post hoc* analyses.

The study inclusion criteria were: (1) ≥18 years of age, (2) admission to the ICU immediately after CPB, and (3) ICU stay of more than one day. The exclusion criteria were: (1) received NaHCO_3_^−^, (2) emergency surgery, (3) pregnancy, (4) chronic renal insufficiency with dialysis, (5) preoperative acute or chronic liver failure, (6) hematologic diseases, (7) central venous catheter dislocation, or (8) death within 48 h. Intraoperative and postoperative ICU management was conducted in accordance with local protocols and international guidelines (Carl et al., 2010; Zhang et al., 2021).

### Data collection and definitions

Intraoperative and ICU management was performed using the same methods as the original study (Zhang et al., 2021). The following data were collected: basic patient characteristics before surgery, intraoperative conditions, postoperative vital signs, and blood test results. The data were collected at: ICU admission (H0), and 4 (H4), 8 (H8), 12 (H12), 24 (H24), and 48 h (H48) after admission. The Acute Physiology and Chronic Health Evaluation II (APACHE II) and sequential organ failure (SOFA) scores were assessed between 24 and 48 h after ICU admission. A blood gas analysis of arteries and veins were performed at ICU admission, and at H4, H8, H12, and H24 using an automated analyzer (ABL800 Flex, Radiometer Medical Aps, Bronshoj, Denmark).

**Figure 1:**
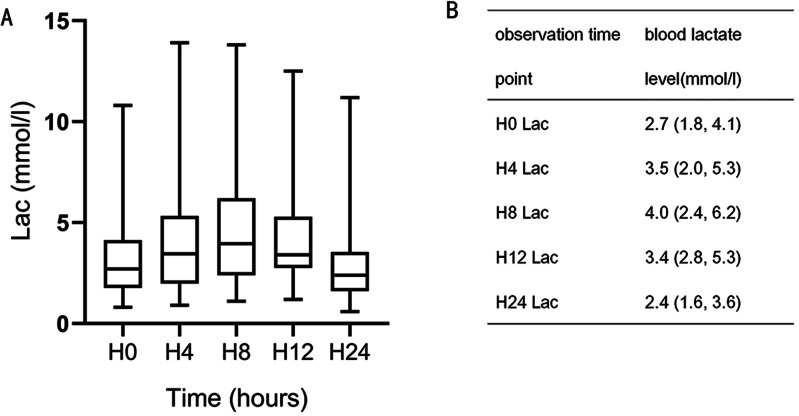
Blood lactate level trends during the 24-h observation period. H8 lactate (Lac), H12 Lac, and H0 Lac were statistically significant (*P* = 0.001, 0.007).

Blood lactate levels gradually increased after CPB, peaking at H8 and then gradually decreasing (Fig. 1). In animal studies, lactic acid level increased after CPB, peaking at 8–12 h and gradually decreasing to the normal range 24 h after surgery (Chiang et al., 2001). A previous clinical study found that lactic acid level peaked 6–9 h after surgery and then gradually decreased, with most levels returning to normal within 24 h (Aktar et al., 2020). Based on these results, H8 was selected for grouping. Patients were divided into three groups according to blood lactate level, HCO_3_^−^, and pH measurement at H8: the lactic acidosis group (LA group; blood lactate levels ≥ 5 mmol/L, pH < 7.35, and HCO_3_^−^ < 20 mmol/L), the hyperlactatemia group (HL group; blood lactate levels > 2 mmol/L, without pH < 7.35 and HCO_3_^−^ < 20 mmol/L), and the normal control group (NC group; blood lactate levels ≤ 2 mmol/L, without pH < 7.35 and HCO_3_^−^ < 20 mmol/L (Kraut & Madias, 2014).

### Outcomes

The primary outcome was the occurrence of MODS at H48. The diagnostic indexes of organ dysfunction used in this study refer to the same criteria as the original study, and MODS was defined as the dysfunction of two or more organs (Zhang et al., 2021). Organ dysfunction within 48 h after surgery was diagnosed in accordance with the following criteria: the diagnostic criteria for acute respiratory distress syndrome (ARDS) was a PaO2/FIO2 of <300 mmHg requiring non-invasive ventilation or invasive mechanical ventilation support; acute kidney injury was defined according to Kidney Disease Improving Global Outcomes (KDIGO): Clinical Practice Guideline for Acute Kidney Injury; additional criteria included total bilirubin levels ≥32 µmmol/L, platelet count <100 × 10^9^/L, and acute tissue hypoperfusion (presence of tachycardia and hypotension, with central venous oxygen saturation (ScvO_2_) level of <65% and cardiac index of ≤2.2 L/min/m^2^), cardiac arrest, arrhythmia (ventricular tachycardia and ventricular fibrillation), and acute neurological dysfunction (stroke, seizure, persistent delirium, and Glasgow coma score below 12).

### Statistical analysis

The statistical analysis was conducted using SPSS 2.0 (IBM Corp., Armonk, NY, USA). The Kolmogorov–Smirnov test was used to evaluate the normality of the continuous variables and the results showed that many continuous variables were non-normally distributed. All continuous variables were expressed as median (range) and analyzed using the Mann–Whitney U-test, the Kruskal–Wallis test (intergroup comparisons), or repeated-measures ANOVA (intragroup comparisons). Categorical variables were presented as m (%) and analyzed using the chi-square test. The factors associated with MODS at H48 were analyzed using a multivariable logistic regression analysis. Variables with *P* < 0.10 in the univariable regression analyses were included in the multivariable regression analysis. GraphPad Prism version 8.0.2 (GraphPad Software Inc., San Diego, CA, USA) was used for the boxplot charts.

## Results

### Clinical characteristics and prognosis of the patients

**Figure 2:**
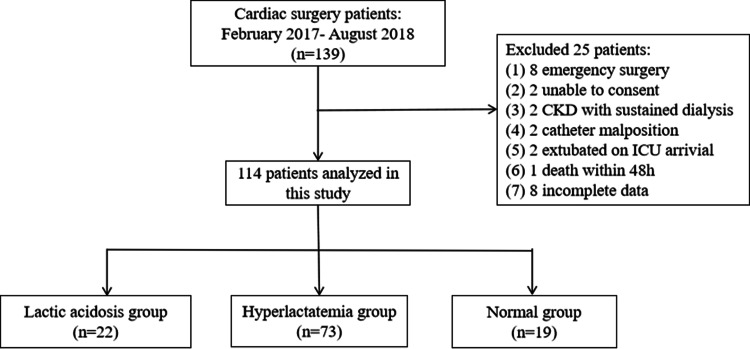
Flowchart. Chronic kidney disease (CKD).

The original study enrolled 139 patients, but 25 were excluded based on the exclusion criteria of the present study, and eight patients were excluded due to incomplete data. Therefore, 114 patients were included in this study: 22 (19%) in the LA group, 73 (64%) in the HL group, and 19 (17%) in the NC group (Fig. 2). There were no significant differences in the preoperative and intraoperative variables among the three groups (all *P* > 0.05). The APACHE II (H24) and SOFA (H24) scores were the highest in the LA group (*P* < 0.05). The ICU stay duration was the longest for the LA group, at 48.5 h (42.5 h, 50 h), compared with 27 h (22 h, 48 h) for the HL group and 27 h (25 h, 46 h) for the NC group (*P* = 0.012). The patients in the LA group had the highest incidence of MODS (36%), compared with 14% in the HL group and 5% in the NC group (*P* = 0.015; *P* < 0.05 for LA *vs.* HL and LA *vs.* N; Table 1).

### Vital signs and perfusion variable at H8

The pH, HCO_3_^−^, and lactate levels were all significantly different between the three groups (all *P* < 0.001). The LA group had lower BE (−7.6 (−9.25, −6.05) *vs.* −2.3 (−4.4, −1.05) *vs.* −1.35 (−2.5, 0.75)), lower SaO_2_-SvO_2_ (21.0 (16.5, 32.5) *vs.* 31.0 (23.75, 37.0) *vs.* 31.0 (29.0, 36.5)) and lower O_2_ER (21.5 (17.05, 32.8) *vs.* 31.3 (24.8, 37.6) *vs.* 31.3 (29.0, 35.4)) compared with the HL and NC groups (all *P* < 0.05). The LA group had higher ScvO_2_ compared with the NC group (76 (66.5, 81) *vs.* 68 (64, 70), *P* = 0.024) (Table 2).

**Table 1:** Patients’ clinical characteristics and prognosis.

**Subject**	LA group (*n* = 22; H8)	HL group (*n* = 73; H8)	NC group (*n* = 19; H8)	*P*
**Preoperative**				
Age (years)	59.50 (55.75, 68.25)	57.00 (48.50, 64.00)	60.00 (50.00, 66.00)	0.421
Sex (male/female)	6/16	34/39	12/7	0.068
Body mass index (kg/m^2^ )	22.59 (19.56, 24.57)	22.77 (20.83, 25.38)	23.07 (20.85, 24.23)	0.633
Smoking (yes/no)	3/19^b^	26/47	10/9	0.029
Diabetes (yes/no)	0/22	6/67	2/17	0.336
Hypertension (yes/no)	8/14	27/46	10/9	0.437
Tumor (yes/no)	1/21	3/70	0/19	0.658
COPD (yes/no)	1/21	1/72	1/18	0.527
Coronary heart disease (yes/no)	4/18	4/69	0/19	0.052
Cerebrovascular disease (yes/no)	2/20	3/70	1/18	0.657
EF (%)	59.5(54,64.25)	63(57,67)	64(53,67)	0.196
pH	7.41 (7.39, 7.42)	7.40 (7.38, 7.42)	7.40 (7.39, 7.41)	0.551
BE (mmol/L)	−0.5 (−1.6, 1.3)	−1.0 (−1.9, 0.5)	1.0 (−0.9, 1.8)	0.351
Hb (g/L)	136 (126, 148)	135 (126, 149)	139 (128, 149)	0.743
PLT count (10^9^ /L)	222 (165, 264)	211 (171, 245)	204 (161, 254)	0.385
ALT (U/L)	25 (14, 36)	20 (12, 40)	25 (16, 35)	0.796
AST (U/L)	22 (20, 27)	22 (20, 31)	29 (24, 33)	0.054
TBIL (µmol/L)	15 (10.5, 16.6)	12.2 (8.6, 17.4)	9.7 (7.5, 16.2)	0.287
DBIL (µmol/L)	4.6 (3.8, 5.6)	4.3 (2.9, 5.8)	3.3 (2.4, 6.0)	0.486
Albumin (g/L)	40 (37.5, 44.6)	42.9 (39.9, 46.3)	43 (39.3, 44.4)	0.165
SCr (µmol/L)	69 (61, 87)	79 (72, 85)	74.5 (61.5, 84)	0.351
BUN (mmol/L)	5.9 (4.6, 8.7)	6.2 (4.8, 7.7)	7.5 (5.8, 8.3)	0.538
GLU (mmol/L)				
**Surgery**				
Valve replacement	14	56	13	0.43
Others	8	17	6	
Duration of surgery (h)	4.5 (3.5, 5.0)	4.0 (3.5, 5.0)	3.6 (3.1, 4.2)	0.354
CBP duration (min)	110 (60, 135)	105 (85, 140)	110 (75, 134)	0.911
Aortic clamping duration (min)	60 (42, 75)	65 (50, 85)	55 (30, 82.5)	0.829
**Prognosis**				
APACHEII (H24) score	10 (9, 13)^a,b^	7 (4,9)	7 (5, 10)	0.003
SOFA (H24) score	7 (5, 8) ^a,b^	5 (3, 7)	5 (4, 6)	0.005
Ventilation duration (h)	16.5 (13.5, 17.5)	16.6 (13.5, 17.5)	17 (13.5, 18)	0.369
ICU stay duration (h)	48.5 (42.5, 50)^a^	27 (22, 48)	27 (25, 46)	0.012
Hospital stay duration (d)	11 (8, 13)	10 (9, 13)	11.5 (8.5, 17)	0.456
Organ dysfunction (yes/no)	11/11	31/42	6/13	0.489
Multiple organ dysfunction (yes/no)	8/14^a,b^	10/63	1/18	0.015

**Notes.**

APACHE II Acute Physiology and Chronic Health Evaluation II ALT alanine aminotransferase AST aspartate aminotransferase BMI body mass index BE base excess BUN Urea nitrogen CPB cardiopulmonary bypass DBIL direct bilirubin H24 24 h after entering the ICU H48 48 h after entering the ICU HB hemoglobin PLT platelet SCr sSerum creatinine SOFA Sequential Organ Failure Assessment TBIL total bilirubin

a *P* < 0.05 between the LA and HL groups.

b *P* < 0.05 between the LA and NC groups.

c *P* < 0.05 between the HL and NC groups.

**Table 2:** Vital signs and perfusion variable at H8.

	LA group (*n* = 22; T8)	HL group (*n* = 73; T8)	NC group (*n* = 19; T8)	P
SBP (mmHg)	100.00 (92.5, 112.5)	106.00 (100.00, 112.25)	98.5 (93.5, 111.25)	0.106
DBP (mmHg)	55.0 (49.5, 60.5)	59.0 (52.75, 64.0)	57.5 (51.25, 63.25)	0.169
MAP (mmHg)	72.0 (63.5, 79.0)	74.0 (68.75, 79.0)	69.5 (67.00, 75.75)	0.196
CVP (mmHg)	10 (7.5, 12.0)	7.0 (6.0, 9.25)	9.0 (7.5, 10.0)	0.033
VIS	4.00 (0.00, 8.00)	3.0 (0.00, 9.00)	8.0 (2.00, 9.00)	0.377
Intake (mL)	1184 (1057.5, 1592.5)	1085 (861.75, 1445)	1150 (860.0, 1551.25)	0.394
Output (mL)	871 (721.5, 1266)^b^	952.5 (843.75, 1226.25)	1242 (768.75, 1707.5)	0.028
Urine volume (mL)	655 (544.0, 1033.5)^b^	782.5 (550.00, 973.75)	1062.5 (653.5, 1367.5)	0.012
Fluid balance (mL)	340.0 (−153.75, 623.5)	33.0 (−187.0, 367.5)	−20 (−395, 185)	0.093
pH	7.29 (7.23, 7.31)^a,b^	7.39 (7.36, 7.43)	7.39 (7.37, 7.44)	<0.001
BE (mmol/L)	−7.6 (−9.25, −6.05)^a,b^	−2.3 (−4.4, −1.05)	−1.35 (−2.5, 0.75)	0.001
HCO_3_ (mmol/L)	19.2 (18.5, 20.5)^a,b^	22.0 (20.3, 23.6)^c^	24.1 (22.4, 25.6)	<0.001
Lactate (mmol/L)	6.8 (5.6, 9.6)^a,b^	4.1 (3.0, 5.4)^c^	1.7 (1.3, 1.9)	<0.001
ScvO_2_ (%)	76 (66.5, 81)^b^	68 (61.0, 74.5)	68 (64, 70)	0.024
SaO_2_-SvO_2_ (%)	21.0 (16.5, 32.5)^a,b^	31.0 (23.75, 37.0)	31.0 (29.0, 36.5)	0.016
O_2_ER (%)	21.5 (17.05, 32.8)^a,b^	31.3 (24.8, 37.6)	31.3 (29.0, 35.4)	0.018
△PCO_2_ (mm Hg)	10.00 (6.50, 11.50)	9.50 (7.75, 11.00)	9.00 (5.00, 11.00)	0.417
△PCO_2_/Ca-vO_2_ (mm Hg/mL)	2.1 (1.8, 3.7)	2.0 (1.6, 2.3)	1.8 (1.3, 2.1)	0.084

**Notes.**

BE base excess CVP central venous pressure Ca-vO_2_ arterial-to-central venous oxygen content difference DBP diastolic blood pressure MAP mean arterial pressure O_2_ER oxygen extraction ratio △PCO_2_ central venous-to-arterial carbon dioxide difference △PCO2/C(a-cv)O_2_ △PCO_2_-to-arterial-to-central venous oxygen content difference rate SBP systolic blood pressure ScvO_2_ central venous oxygen saturation SaO_2_-SvO_2_ arterial-to-central venous oxygen saturation difference VIS vasoactive-inotropic score

a *P* < 0.05 between the LA and HL groups.

b *P* < 0.05 between the LA and NC groups.

c *P* < 0.05 between the HL and NC groups.

### Risk factors associated with MODS

In the univariable analyses, patient age (OR = 1.054, 95% CI [1.003–1.109], *P* = 0.038), the LA group (*vs.* NC, OR = 10.286, 95% CI [1.148–92.185], *P* = 0.037), and ΔPCO_2_ at H8 (OR = 1.197, 95% CI [1.022–1.401], *P* = 0.025) were associated with MODS after CPB (Table 3).

**Table 3:** The risk factors of MODS were analyzed, and the perfusion index at the H8 time point was analyzed by binary logistic regression.

Subject	Univariate	
	OR (95% CI)	P
Group		
NC	Reference	
HL vs. NC	2.857 (0.342–23.835)	0.332
LA vs. NC	10.286 (1.148–92.185)	0.037
Age	1.054 (1.003–1.109)	0.038
ScvO_2_ (H8)	0.953 (0.908–1.000)	0.051
O_2_ER (H8)	1.023 (0.995–1.052)	0.108
△PCO_2_ (H8)	1.197 (1.022–1.401)	0.025
△PCO_2_/Ca-vO_2_ (H8)	1.218 (0.849–1.746)	0.283

**Notes.**

*P* < 0.05 was considered statistically significant.

MODS multiple organ dysfunction syndrome OR odds ratio CI confidence interval NC normal control HL hyperlactatemia LA lactic acidosis ScvO_2_ central venous oxygen saturation SaO_2_-SvO_2_ arterial-to-central venous oxygen saturation difference O_2_ER oxygen extraction ratio △PCO_2_ central venous-to-arterial carbon dioxide difference △PCO2/C(a-cv)O_2_ △PCO_2_-to-arterial-to-central venous oxygen content difference rate

## Discussion

This *post hoc* analysis of a previous prospective study aimed to determine the association between LA and the occurrence of MODS after CPB. The LA group had long ICU stays and a high incidence of MODS, suggesting that LA was associated with MODS after CPB.

Tissue hypoperfusion and lactic acid accumulation can occur during cardiac surgery (Chiang et al., 2001; Kanazawa et al., 2015; Naik et al., 2016; Teloh et al., 2017). When tissue perfusion is restored after CPB, lactic acid displays a washout effect, and a large amount of lactic acid is released into the blood, leading to apparent hyperlactatemia, but that lactic acid is cleared rapidly if the heart and vital organs recover well after surgery, preventing LA (Kanazawa et al., 2015). LA occurs when the body fails to compensate (Kanazawa et al., 2015). Some studies suggest that high lactacidemia during operation is related to CPB time and aortic occlusion time (Naik et al., 2016; Ranucci et al., 2006). In this study, lactic acid levels were grouped eight hours after surgery, and there was no difference in cardiopulmonary bypass time and aortic occlusion time among the three groups. This may be because the patients were stable before surgery and the operations were performed by the same surgeon, so there was no difference in time of CPB and aortic occlusion. Naik et al. (2016) reported that the incidence of hyperlactatemia (blood lactate levels ≥ 4 mmol/L) was 42.7%, and these patients had an increased incidence of postoperative atrial fibrillation and extended ICU stays. Renew et al. (2016) observed that severe hyperlactatemia (blood lactate level > 10 mmol/L) was associated with increased mortality. Zante et al. (2018) reported that blood lactate levels >4.0 mmol/L, combined with a BE <6.7 mmol/L, could predict mortality, whereas blood lactate levels >4.0 mmol/L alone did not affect mortality after cardiac surgery. However, these studies ignored the presence of lactic acidosis in patients with severe hyperlactacemia, especially when the blood lactate levels were >5 mmol/L. In the present study, the blood lactate levels were 6.8 (5.6, 9.6) mmol/L in the LA group and 4.1 (3.0, 5.4) mmol/L in the HL group. The LA group tended to be severely ill, had a prolonged ICU stay, and a high incidence of MODS. Hyperlactemia alone did not affect prognosis.

Clinically, ScvO_2_, O_2_ER, ΔPCO_2_, and ΔPCO_2_/Ca-vO_2_ are often used to evaluate tissue perfusion (Janotka & Ostadal, 2021). The O_2_ER is the amount of oxygen taken out of the blood by the tissues. Normal O_2_ER is about 25%–30%. In this study, O_2_ER was calculated as 1-ScvO_2_/SaO_2_. In sepsis, because oxygen free radicals and inflammatory mediators damage the structure of the mitochondria, the number and function of mitochondria declines. The longer severe sepsis lasts, the more irreversible mitochondrial structure and function changes occur, resulting in mitochondrial dysfunction and microcirculation disorders, decreased oxygen use, decreased oxygen consumption, increased ScvO_2_, and reduced O_2_ER (Gong & Li, 2013; Pope et al., 2010). In a study of patients with septic shock, the hospital mortality rate was 41.7% in the low O_2_ER group, which was higher than the normal and high O_2_ER groups. O_2_ER was negatively correlated with ScvO_2_ (*R*^2^ = 0.878, *P* < 0.001), and high ScvO_2_ combined with low O_2_ER was associated with mitochondrial dysfunction (Park et al., 2015). In the present study, there was no difference in mean arterial pressure between the three groups, but the LA group had decreased urine volume, increased ScvO_2_, and decreased O_2_ER, due to the SIRS produced by CPB, and the oxygen free radicals and various chemia-reperfusion injuries leading to mitochondrial dysfunction, causing lactic acidosis (Balzer et al., 2015; Janotka & Ostadal, 2021; Stephens et al., 2020). Dekker et al. (2019) found persistent microcirculation perfusion disorders through hypoglossal flow dark field microcirculation imaging technology in the first three days after CPB. A separate study found that microcirculation disorders persisted 24 h after CPB through the reduction of functional capillary density (FCD), perfusion vessel density (PVD), or perfusion vessel proportion (PPV), even when general circulation was stable (den Os et al., 2020). A single-center study analyzed the relationship between microcirculation heterogeneity and hemodynamics and found that SaO_2_-SvO_2_ and O_2_ER decreased in microcirculation perfusion disorders after CPB (Koning et al., 2014). In the present study, there were high ScvO_2_ and low O_2_ER in the LA group, suggesting that LA after CPB may be accompanied by microcirculation and mitochondrial dysfunction. MODS can be the result of both hypoperfusion and hypo-oxygenation (Rixen & Siegel, 2005), or the result of microcirculation and mitochondrial dysfunction, reflected by LA (Garcia-Camacho et al., 2020; Greenwood et al., 2021; Teloh et al., 2017). Even if the patient’s circulation is stable after CPB, there might be mitochondrial dysfunction and microcirculation disorders, resulting in organ dysfunction. However, this study was a retrospective analysis, and the indicators of mitochondrial function could not be analyzed with LA. Therefore, future studies are needed to evaluate this relationship.

In this study, patient age and △PCO2 were risk factors for MODS, but the OR value of age and △PCO2 were close to 1, and were of little clinical significance. The research team previously found that ΔPCO2 and ΔPCO2/Ca-vO2 levels are not reliable predictors of organ dysfunction occurrence at H48 (Lefering et al., 2013; Zhang et al., 2021). LA is a prognostic factor for MODS in sepsis (Bakker et al., 1996; Doshi et al., 2018; Gattinoni et al., 2019), trauma with massive hemorrhage (Lefering et al., 2013), and cardiogenic shock (Jentzer et al., 2022). The occurrence of MODS leads to longer ICU stays, higher hospital expenses, and increased morbidity and mortality (Asim, Amin & El-Menyar, 2020; Zhao et al., 2022). In this study, LA was associated with MODS. However, due to the insufficient number of cases in this study, no further multi-factor analysis was done. Because LA can be detected quickly through a blood gas analysis, LA could be used as a monitoring indicator of MODS after CPB. There are many causes of lactic acidosis, including tissue hypoperfusion, hyperglycemia, and endogenous catecholamines caused by the stress response during cardiac surgery. The liver accounts for 60% of lactic acid metabolism, and the kidney accounts for 30% (Lefering et al., 2013; Stephens et al., 2020). Lactic acidosis could be avoided by controlling blood glucose and body temperature, shortening the time of aortic blood flow blockade during operation, optimizing volume management, supporting organ function treatment, and ensuring the perfusion of macrocirculation and microcirculation after operation. LA could be used as a reference index for the occurrence of MODS after CPB, better guiding clinical treatment for post-surgical patients.

## Limitations

This study has some limitations. First, this was a *post hoc* analysis of a completed prospective study. The sample size of the original study was limited, and some patients had to be excluded from the present analysis. This small sample size likely led to the wide 95% CI observed in the association between LA and MODS in the univariate analysis. Therefore, no further multivariate regression analysis was performed. Second, it was a single-center study, and generalizability might be limited. Third, this study did not include data on whether LA after cardiac surgery was only caused by increasing lactic acid levels or by other factors. Therefore, a future prospective multicenter study is planned, with a larger sample size, to collect relevant data with MODS as the outcome indicator, monitoring mitochondrial function indexes and investigating the relationship between LA and MODS.

## Conclusion

This study identified a correlation between lactic acidosis and MODS after CPB. LA is an easy-to-obtain clinical indicator, so LA should be closely monitored after CPB to help improve patient prognosis after CPB.

## Supplemental Information

10.7717/peerj.16769/supp-1 Supplemental Information 1 Raw data

## Additional Information and Declarations

### Data Availability

The following information was supplied regarding data availability: The raw data are available in the Supplementary File.
